# Nutritional value of the Middle Eastern diet: analysis of total sugar, salt, and iron in Lebanese traditional dishes

**DOI:** 10.12688/f1000research.26278.1

**Published:** 2020-10-19

**Authors:** Maha Hoteit, Edwina Zoghbi, Mohamad Al Iskandarani, Alissar Rady, Iman Shankiti, Joseph Matta, Ayoub Al-Jawaldeh

**Affiliations:** 1 Faculty of Public Health, Lebanese University, Beirut, Lebanon; 2 Country Office for Lebanon, World Health Organization, Beirut, Lebanon; 3 Industrial Research Institute, Lebanese University, Beirut, Lebanon; 4 Department of Nutrition, Faculty of Pharmacy, Saint Joseph University, Beirut, Lebanon; 5 Regional Office for the Eastern Mediterranean, World Health Organization, Cairo, Egypt

**Keywords:** Total sugar, salt, iron, Lebanese traditional dishes, governorates

## Abstract

**Background:** The expanding burden of diet-related non-communicable diseases in the Eastern Mediterranean Countries requires urgent public health vigilance and actions. This study aimed at establishing a database analysis of total sugar, salt and iron content in Lebanese foods, focusing on traditional dishes.

**Methods:** The collection of food samples was done using stratified sampling techniques. These samples were classified into five strata, taking into account variation by geographical area (Mount Lebanon, Bekaa, Beirut, Tripoli, and Saida). The number of samples per governorate was estimated to be 30 according to the variability in the dishes' composition. Food samples were chemically analyzed for total sugar, salt, and iron.

**Results:** Among all the governorates, all the tested traditional Lebanese dishes contained little total sugar. More than 60% of the samples tested were rich in sodium. The sodium content ranges were 120-720 mg/100 g in Mount Lebanon, 240-960 mg/100 g in Bekaa, 80-520 mg/100g in Beirut, 252-1952 mg/100g in Tripoli and 40-680 mg/100 g in Saida. The highest mean amount of sodium was observed in the dishes *Fatayer Sabanikh* and *Malfouf Mehche* (≥ 600 mg/100 g). Furthermore, more than 80% of the samples had poor amounts of iron in all governorates.

**Conclusion:** This study emphasizes the need for multi-cultural education and awareness on food sources of salt and iron, and the health effects regarding high intake of salt and low intake of iron. This study is a stepping stone for further research exploring total sugar, salt and iron content of traditional dishes, as well as potential intake by individuals in the Lebanese population.

## Introduction

In light of global spread of non-communicable diseases (NCDs), accounting for 70% of the 41 million deaths each year, the probability of premature death, in 2018, caused by NCDs is 91% of all deaths in Lebanon ^1^. The global rise in the prevalence of NCDs is a consequence of shifting dietary patterns specified by a high intake of meals rich in fat, sugar, salt, and low in fiber and micronutrients ^2^. Home cooking and at-home eating have become scarce, while processed foods and prepared meals are increasing and have become a major part of the population's lives ^2^. Furthermore, it is widely believed that urbanization has played a role in producing side effects for NCDs-related health outcomes ^3^. Cardiovascular diseases (CVDs) constitute the primary cause of death in the Middle Eastern Region ^4^. According to the World Health Organization (WHO), based on 2017 data, 17.9 million people die each year from CVDs, representing 31% of all deaths worldwide ^5^. Furthermore, around 23.6 million people are expected to die from many forms of CVDs by 2030 ^6^.

High blood pressure is a dominant factor for CVDs ^7^. A higher intake of dietary salt (equivalent to 5 g salt/day) is associated with high levels of blood pressure, cardiovascular diseases and strokes ^5^. In May 2013, the WHO Member States decided to target a reduction in the global intake of salt by 30% by 2025 ^8^. A systematic review, published in 2020, on 133 randomized control trials showed a reduction of 1.10 mmHg in systolic blood pressure and 0.33 mmHg reductions in diastolic blood pressure with every 50 mmol reduction in 24-hour sodium excretion. This can lead to a protection against vascular complications and decrease CVD risk ^9^. In addition, there is considerable evidence of the advantages of Mediterranean diet on health ^10^. An umbrella review and meta-analyses of observational and randomized controlled trials showed a strong relation between the adherence to Mediterranean diet and the 50% reduction in mortality from NCDs ^10^.

Today, the major target of nutrition interventions is the energy dense "added sugars". The WHO definition for added sugars is "all monosaccharides and disaccharides added to foods and beverages by the manufacturer, cook, or consumer, and sugars naturally present in honey, fruit juices, and fruit concentrates" ^11^. In this definition the "total sugar" term that is "naturally occurring or intrinsic sugars, which are stored within the cells of intact fruits, vegetables, or involve lactose in milk or unsweetened dairy products" was not included ^11^. In 2015, the WHO recommended the reduction of the daily intake of added sugars to less than 10% of the total energy intake ^12^. Additional suggestion of reducing the daily intake of added sugars to less than 5% of total energy intake may promote additional health benefits ^12^. Until today, total sugar has no recommendation for the daily intake ^13^. However, for the aim of achieving the goals set by the *Global Action Plan for NCDs 2013–2020*, a guideline on total sugar was developed to help in the reduction of the prevalence of diabetes and obesity, and decrease the incidence of premature deaths caused by NCDs by 25% by 2025 ^14^.

Furthermore, deficiencies in iron lead to the development of anemia, which is one of the most common nutrition problems. As per the WHO, more than 2 billion people are anemic and iron deficiency is one of the main causes ^15^. In addition, there is a high prevalence of 42% of children less than 5 years of age and more than 40% of pregnant women who are anemic ^15^. In 2012, specific dietary guidelines for Arab countries were developed by the WHO and the Arab Center for Nutrition. Only seven countries that represent 29% of the Eastern Mediterranean Region population (namely Afghanistan, KSA, Oman, Lebanon, Qatar and Iran) designated their national food based dietary guidelines ^16, 17^.

The main purpose of this study is to initiate a database analysis of total sugar, salt and iron content in Lebanese foods, focusing on traditional dishes.

## Methods

### Dish selection

The definition of ‘composite dishes’ is "dishes consumed at main meals (i.e. lunch or dinner), whose preparation involves culinary skills and contains ingredients from at least three of five main food groups: meat/poultry/fish and eggs; dairy products; fruits and vegetables; starchy foods including legumes; added sweets and fats" ^18^. The list of Lebanese composite dishes frequently consumed by Lebanese citizens was retrieved from a study done in 2005 on a representative sample of 799 Lebanese adults ^18^, and in line with a study conducted in 2009 where the objective was to compare the consumption of traditional dishes between Lebanon and France ^19^. The Lebanese diet includes a range of foods with often complex recipes, and it is rarely possible to analyze all the types of dishes. In such cases, a laboratory analysis of the traditional dishes and a calculation of some nutrients should be achieved. The names of the dishes most eaten by Lebanese citizens and chosen for this study are shown in Table 1.

**Table 1.** Local names and main ingredients of selected traditional dishes frequently consumed in Lebanon.

Appetizer/Dish	Ingredients
**Baba Ghanouj**	Aubergines, garlic cloves, lemon juice, tahini, pomegranate seeds, salt
**Batata mihchi**	Lamb ground, onions, butter, salt, pepper, pine nuts, potato, tomato juice
**Bulgur b banadoura**	Coarse bulgur wheat, small pearl onions, chickpeas, cinnamon stick, caraway seed, vegetable oil, mild white pepper, salt
**Chichbarak**	Chichbarak Dough: multi-purpose flour, salt, water warm to form a paste, yeast, sugar. Meat Stuffing: ground beef, salt to taste, black pepper to taste, cinnamon powder to taste, onion finely chopped, pine nuts, olive oil, bushel of parsley chopped Chich Barak Stew: yogurt, water, starch, garlic cloves crushed (optional), rice, dried mint, salt to taste
**Falafel**	Dry peeled fava beans dried chickpeas (aka Garbanzo beans), Italian parsley (chop away the stems), green cilantro (chop away the stems), freshly peeled crushed garlic cloves, red or yellow onion, green onions, salt, black pepper, flour, baking soda, red chili pepper (optional, if spicy falafel is desired), cumin, coriander. Falafel Tahini Sauce: Tahini paste, freshly squeezed lemon juice, garlic cloves, crushed, salt
**Fatayer Sabanikh**	Fresh spinach, onions, pine nuts, lemon juice, olive oil, sumac, salt, plain white flour, caster sugar, baker yeast, olive oil, salt
**Fatteh**	Chickpeas, tomatoes, onion, basil leaves, garlic cloves, pitta bread, pine nuts, yogurt, tahini and vinegar, vegetable oil, salt and pepper
**Fattoush**	Lettuces or romaine lettuce, cherry tomatoes, cucumbers, radishes, spring onions, flat-leaf parsley mint, pitta bread, olive oil, vinegar, sumac, salt
**Foul moudamas**	Broad beans, baking soda, water, water, salt, garlic cloves minced, lemon juice, olive oil
**Hindbe b zet**	Chicory greens, water, olive oil, onions, salt, lemon juice
**Hummus bi tahini**	Chickpeas, garlic cloves, lemon juice, Tahini, olive oil, salt
**Kafta w batata**	Minced lamb, flat-leaf parsley, onions, salt and pepper, onions, red pepper, tomato juice, debs roman, olive oil, salt and pepper, potatoes, ripe tomatoes, vegetable oil
**Kibbeh b sayniye**	Finely ground beef (or lamb, lean), bulgur cracked wheat, salt, all spice,cumin, onions (finely chopped)
**Koussa Mihchi**	Minced lamb, small zucchini, short grain rice, olive oil, salt and black pepper
**Lahm bi ajeen**	Plain white flour, caster sugar, baker's yeast, salt, olive oil, minced lamb, tomatoes, few drops of pomegranate molasses, salt and pepper
**Loubieh b zet**	Vegetable oil, white onions, sliced, frozen bag green beans, garlic cloves, peeled, Cans of chopped tomatoes, salt and sweet pepper to taste, 7 spices, extra-virgin olive oil
**Malfouf mehche**	Cabbage leaves, basic vegetables stuffing, tomato, lemon juice, water, cinnamon, garlic cloves, dry mint
**Moujadara**	Green or coral lentils, short-grain rice, onions, olive oil, salt
**Moghrabieh**	Dry dough, chickpeas, pearl onions, vegetable oil, caraway butter, ground cinnamon, ground cumin, salt, black pepper
**Mousaka batinjen**	Aubergine,yellow or white onion, diced, garlic cloves, minced, low-salt chickpeas, extra virgin olive oil, low-salt diced tomatoes, tomato paste, piquant post spicy mint blend, Pita chips or crusty bread for dipping, salt and pepper to taste
**Riz a djeij**	Chicken breast, basmati rice, carrot, onions, tomato juice, whole black peppercorns, whole green cardamoms, cinnamon, cloves, cumin, vegetable oil, salt
**Riz b lahme**	Medium fat meat, basmati rice, carrot, onions, tomato juice, whole black peppercorns, whole green cardamoms, cinnamon, cloves, cumin, vegetable oils, salt
**Sayadieh**	Sea bass, scaled and gutted or in fillets, basmati rice, onions, caraway seeds, ground cumin, pines nuts, olive oil, fish stock, vegetable oil, salt, flour, butter, lemon juice
**Shawarma Djeij**	Chicken, olive oil, onions, red vinegar, lemon juice, pepper, cinnamon, nutmeg, salt, cloves of garlic
**Shawarma Lahme**	Meat, olive oil, onions, red vinegar, lemon juice, pepper, cinnamon, nutmeg, salt, cloves of garlic
**Tabboule**	Tomatoes, spring onions, flat leaf parsley, mint, bulgur wheat, lemon juice, olive oil, salt
**Warak enab**	Vine leaves, tomatoes, onion, flat-leaf parsley, mint, lemon juice, short-grain rice, meat, olive oil, salt
**Yakhnet Bemieh**	Lamb cubed, onions, garlic cloves minced, green coriander, okra, lemon juice, salt, pepper, water and tomatoes
**Yakhnet Fassoulia**	Shoulder of lamb, fresh white haricot beans, coriander, onions, garlic cloves, tomato juice, olive oils, water or chicken stock, salt and black pepper
**Yakhnet Mloukhie**	Free-range chicken, basmati rice, coriander, garlic cloves, onion, shallots, pitta bread, vinegar, lemon juice, vegetable oil and salt

### Data collection

Samples of cooked composite dishes were collected (see below). The food samples were bought from five different governorates (Mount Lebanon, Bekaa, Beirut, Tripoli and Saida), taking into account geographical and cultural variations.

A total of 500 g of each dish was collected and used for analysis. According to Greenfield and Southgate, this size is a convenient sample to avoid errors during analysis ^20^.

Our research group collected 500 g of 30 types of traditional dishes from five different central kitchens in the following governorates: Mount Lebanon, Beirut, Bekaa, Tripoli, and Saida to show wide representation. Thus, 150 samples were collected. A list of kitchens that served tradition meals, found on internet, was prepared for each of the governorates. The central kitchens were randomly chosen from the results of the internet search, based on the following criteria: 1) their specialty in cooking home-made dishes; 2) their popularity; and 3) their involvement in supporting women as part of the social entrepreneurship initiatives that are aimed at empowering women.

### Chemical analysis

After the receipt of food samples, 500 g of each composite dish was mashed, and then analyzed in the laboratory. The remaining samples were kept frozen at -18°C in tight containers for further analysis.

***Total sugar*.** An amount ranging between 2 and 10g (depending on expected value of sugar content) of the sample was put in a 250 ml volumetric flask. 125 ml of 50% alcohol solution was placed in steam bath overnight. The volume was made up to 125 ml with 95 % ethanol followed by filtration with filter paper. 100 ml of the filtrate was pipetted into a beaker and evaporated to reduce the volume to 20 – 30 ml. After that, the solution was placed in 100 ml volumetric flask and rinsed thoroughly with water and then added to the flask. Then the solution was made up to volume of 100 ml and mixed thoroughly. Later on, 50 ml of the obtained solution was placed in 100 ml volumetric flask,then a piece of Lithmus paper was added and the total was neutralized with hydrochloric acid (HCL). An addition of 5 ml of HCl was added and the inversion of sucrose was done at room temperature. When the inversion of sucrose was complete, the solution was transferred to a beaker and neutralized with Na _2_CO _3_ until a pink color appeared. Later, the solution was returned to 100 ml flask, diluted to volume of 100 ml with water. Filtration was done when necessary, and the determination of reducing sugars in 50 ml of the solution was done by adding 25 ml each of Fehling A and Fehling B solutions and boiling for 4 min. After a red precipitate formed, the solution was cooled and filtered with a Gooch crucible. The mixture was dried in oven and the calculation of the precipitate was achieved (AOAC 906.03, 930.36 and 975.15) ^21^.

The content of reducing sugars was red from the table, and calculated as % sugars using the formula

$$\text{\%}\;\text{sugar}\;\text{=}\;\frac{\text{Reading}\;\text{from}\;\text{table}\;\text{×}\;\text{dilution}\;\text{factors}\;\text{×}\;100}{1000\;\text{×}\;\text{wt}\;\text{sample}}$$

***Salt*.** To measure the salt content, 2–3 g of the food sample was weighteded into a pre-weighed furnace-proof crucible. It was baked until ash in a 600° C furnace overnight.

When the sampled had cooled, the ash was dissolved in water and was transferred quantitatively in a 50 ml volumetric flask. After making up to volume of 50 ml, the solution obtained was transferred into an Erlenmeyer flask. Later on, 1 ml of potassium chromate indicator solution was added and the solutions was titrated drop wise with the addition of 0.1 N silver nitrate solutions until the color of the solution changed to a reddish brown. A blank test was carried out in parallel using 50 ml of distilled water instead of the sample solution. The blank value did not exceed 0.2 ml of silver nitrate (AOAC 937.09 & ISO 9297) ^21^.

The chloride content in the sample was calculated using the formula.

$$\text{Chloride}\;\text{content}\;\text{=}\;\frac{\text{Vs}\;\text{×}\;\text{N}\;\text{×}\;58.5\;\text{×}\;100}{\text{Wt}\;\text{×}\;1000}$$

Where Vs = volume in ml of silver nitrate

N = normality of silver nitrate

Wt = weight of sample used

Finally, the chloride content was used to calculate NaCl. The molecular weight of NaCl is 58.44 and the molecular weight of Chloride is 35.453 g. Thus, to calculate the NaCl in the solution, the Cl should be multiplied by 1.65 (58.44 divided by 35.453).

***Iron*.** An amount of 15 g of the edible portions was dried in an air oven at 105°C for more than 2 hours then charred until smoke disappearance. A muffle furnace was used to bake the charred sample at 550°C until a grey-colored ash yielded. 25–30 ml of 3.7% HCl was used to treat the ash, and then the mixture was conveyed to a container and done up to a volume of 50 ml with HCl. For each tested food, two solutions of ash were made ready to be analyzed twice. Each fraction of the ash sample was used for iron measurement using a Varian Atomic Absorption Spectrophotometer model 175 with an air-acetylene flame. Ferric nitrate solution was used as a standard. For each ash solution, at least three readings were obtained, and the average was calculated based on AOAC 975.03, 985.35 &965.09 ^21^.

All the above chemical analyses were conducted at the Industrial Research Institute (Beirut, Lebanon), which is an accredited laboratory for chemical analyses. After food samples were analyzed, stamped laboratory results were received and data was entered on Excel 2019. The data validation process was conducted, and cross checked by two research assistants.

### Recipes

The recipes of the dishes in this study were selected from those mostly consumed in Lebanon. The dishes were bought and analyzed as they are consumed by customers. The description (ingredients) of the selected recipes is presented in Table 1.

## Results and discussion

The composition of 100 g of the Lebanese traditional dishes in the five Lebanese governorates is shown in Table 2.

**Table 2.** Analysis of total sugar, salt and iron in 100 g of traditional dishes from five governorates in Lebanon. Units: Total sugar (Tot S.) in gram, salt (NaCl) in gram, sodium (Na) in milligram, and iron in milligram.

Appetizer/Dish	Amounts in 100 g of edible portions																			
	Mount Lebanon				Bekaa				Beirut				Tripoli				Saida			
	Tot S.	NaCl	Na	Iron	Tot S.	NaCl	Na	Iron	Tot S.	NaCl	Na	Iron	Tot S.	NaCl	Na	Iron	Tot S.	NaCl	Na	Iron
**Baba Ghanouj**	2.8	0.7	280	0.6	1.8	1.2	480	0.7	2.1	0.9	360	0.9	3	0.6	252	0.6	1.5	0.9	360	0.7
**Batata mihchi**	2.5	1.1	440	1.1	0.4	0.8	320	1.1	1.5	0.7	280	0.7	1.5	1.1	476	1.1	0.8	1.3	520	1.2
**Bulgur b banadoura**	1.5	1.4	560	1.5	1.6	0.8	320	1.6	1.3	1.3	520	1	1.4	1.2	500	1.2	1.8	1.1	440	1.3
**Chichbarak**	2.3	0.7	280	2.8	2.7	1.1	440	2.7	2.5	0.7	280	0.7	6	1.1	456	1.1	2.6	1	400	2.5
**Falafel**	3.6	1.4	560	1.8	3.2	1.9	760	1.7	2	1.3	520	3.2	3	1.7	700	1.7	4.3	1.4	560	1.9
**Fatayer Sabanikh**	1.6	0.9	360	5	2.4	1.5	600	5.1	1.4	0.7	280	4.7	2.3	4.8	1952	4.8	2.2	0.9	360	4.5
**Fatteh**	3	0.6	240	0.9	2.2	0.6	240	1.7	3.2	0.6	240	1.2	3.4	1.3	528	1.3	0.6	0.1	40	1
**Fattoush**	1.6	0.3	120	0.6	1.3	1.3	520	0.8	1.8	0.4	160	2.2	5.2	0.9	380	0.9	2	0.7	280	0.4
**Foul moudamas**	1	1	400	0.6	1	2.4	960	0.9	3.3	0.7	280	0.7	1.1	0.6	272	0.6	2.3	1.4	560	0.7
**Hindbeb zet**	4.2	0.7	280	1.6	1.3	0.9	360	2	2.3	0.8	320	2.6	4.6	1.7	688	1.7	0.7	0.9	360	1.7
**Hummus bi tahini**	1.6	0.8	320	0.8	2.7	0.6	240	1.2	2.4	0.8	320	1.2	3	1	400	1	1.8	0.9	360	0.9
**Kafta w batata**	1.1	1.2	480	4.1	0.9	0.9	360	1.3	2	0.8	320	1.4	1.9	1.2	500	1.2	1.3	1.2	480	3.9
**Kibbeh b sayniye**	2.5	1.2	480	2	2	1	400	2	3.3	0.8	320	1.3	2.5	1.8	748	1.8	1.3	1.1	440	1.9
**KoussaMihchi**	0.4	1.2	480	1.3	0.8	0.9	360	1.1	1.1	0.7	280	1.7	1.9	1.7	704	1.7	0.8	1.2	480	1.2
**Lahm bi ajeen**	1.5	0.5	200	1.9	4.7	0.7	280	3.1	3.4	0.7	280	1.7	4.1	1.3	528	1.3	3.2	0.7	280	2
**Loubieh b zet**	1.8	0.6	240	0.8	0.6	0.8	320	1.1	1.8	0.9	360	0.9	0.9	2	820	2	0.9	0.6	240	0.7
**Malfouf mehche**	2.1	1.8	720	1	1.6	0.7	280	1	2.1	0.9	360	1.1	2.6	2.7	1088	2.7	1.6	1.7	680	1.1
**Moujadara**	1.5	0.4	160	1.2	1	0.9	360	1.1	2	0.5	200	1.4	1.1	1.6	664	1.6	1.6	0.6	240	1.3
**Moghrabieh**	1.2	0.4	160	0.9	1.5	1	400	1.2	1.2	0.2	80	1	1.7	0.9	364	0.9	1.8	0.6	240	0.8
**Mousaka** **batinjen**	2	0.6	240	1	1.5	1.9	760	2.4	0.5	1.3	520	0.9	1.9	1.1	456	1.1	1.9	0.7	280	0.9
**Riz a djeij**	1.2	0.7	280	1	0.7	0.7	280	3.8	0.4	1	400	1	0.2	1.2	500	1.2	1.3	0.4	160	0.9
**Riz b lahme**	1	0.4	160	1.3	0.6	0.9	360	1.7	1.2	0.9	360	1	2	1	436	1	0.9	1	400	1.2
**Sayadieh**	0.4	0.8	320	1.3	0.8	1.5	600	1.4	0.2	0.8	320	0.7	0.6	1.2	500	1.2	0.7	1.4	560	1.1
**Shawarma Djeij**	2.2	1.4	560	1.6	2	1.2	480	1.5	1.8	0.8	320	1.5	1.9	1.3	544	1.3	0.1	0.8	320	1.5
**Shawarma** **Lahme**	0.9	0.7	280	1.5	0.9	1.4	560	1.4	1.1	0.4	160	1.3	1.1	1.7	700	1.7	1	0.9	360	1.3
**Tabboule**	0.6	1.1	440	1	1.3	1.9	760	1.2	1.4	0.6	240	2.3	1.3	1.5	600	1.5	0.8	0.7	280	0.9
**Warak enab**	1.1	1.3	520	1.3	1	0.9	360	1.7	2.5	0.7	280	1	0.8	1	400	1	0.3	1.3	520	1.4
**Yakhnet Bemieh**	1.4	1.1	440	1.5	5.5	0.8	320	1	1.7	0.9	360	1.3	2.2	1.5	600	1.5	1.6	0.7	280	1.4
**Yakhnet** **Fassoulia**	1	1	400	0.9	0.9	0.7	280	1.1	1	0.6	240	1.8	1.1	1.6	668	1.6	0.9	0.9	360	0.8
**Yakhnet** **Mloukhie**	0.9	0.9	360	1	0.8	0.9	360	1.1	0.2	0.7	280	1	0.9	1.6	668	1.6	0.8	0.9	360	1.1

### Total sugar

Among all the governorates, all the tested traditional Lebanese dishes contained little total sugar. The highest total sugar (≥3 g/100 g) was observed in the dishes *Hindbe b bzet* and *Falafel* from Mount Lebanon, in *Yakhnet Bemieh* and *Lahm bi ajeen* from Bekaa, in *Lahm bi ajeen* and *Foul Moudamas* from Beirut, *Lahm bi ajeen* and *Fattoush* from Tripoli, and in *Lahm bi ajeen* and *Falafel* from Saida ( Table 2). As per Table 3, *Lahm bi ajeen* and *Falafel* ranked first in the highest mean total sugar.

**Table 3.** The mean values from the five governorates of total sugar, salt and iron in the traditional dishes. Units: Total sugar in gram, salt in gram, sodium in milligram and iron in milligram.

Appetizer/Dish	Mean values in 100 g			
	Total sugar	Salt	Sodium	Iron
**Baba Ghanouj**	2.2	0.8	346.4	0.7
**Batata mihchi**	1.3	1	407.2	1
**Bulgur b banadoura**	1.5	1.1	468	1.3
**Chichbarak**	3.2	0.9	371.2	2
**Falafel**	3.2	1.5	620	2.1
**Fatayer Sabanikh**	1.9	1.7	710.4	4.8
**Fatteh**	2.4	0.6	257.6	1.2
**Fattoush**	2.3	0.7	292	1
**Foul moudamas**	1.7	1.2	494.4	0.7
**Hindbe b zet**	2.6	1	401.6	1.9
**Hummus bi tahini**	2.3	0.8	328	1
**Kafta w batata**	1.4	1	428	2.4
**Kibbeh b sayniye**	2.3	1.1	477.6	1.8
**Koussa Mihchi**	1	1.1	460.8	1.4
**Lahm bi ajeen**	3.3	0.7	313.6	2
**Loubieh b zet**	1.2	0.9	396	1.1
**Malfouf mehche**	2	1.5	625.6	1.4
**Moujadara**	1.4	0.8	324.8	1.3
**Moghrabieh**	1.4	0.6	248.8	1
**Mousaka batinjen**	1.5	1.1	451.2	1.3
**Riz a djeij**	0.7	0.8	324	1.6
**Riz b lahme**	1.1	0.8	343.2	1.3
**Sayadieh**	0.5	1.1	460	1.1
**Shawarma Djeij**	1.6	1.1	444.8	1.5
**Shawarma Lahme**	1	1	412	1.4
**Tabboule**	1	1.1	464	1.4
**Warak enab**	1.1	1	416	1.3
**Yakhnet Bemieh**	2.4	1	400	1.4
**Yakhnet Fassoulia**	0.9	0.9	389.6	1.2
**Yakhnet Mloukhie**	0.7	1.0	405.6	1.2

### Salt

The sodium (Na) level was high in most dishes ( Table 2). The highest mean amount of Na was observed in *Fatayer Sabanikh* and *Malfouf Mehche* (≥ 600 mg/100 g) ( Table 3). The Na content ranges are 120–720 mg/100 g in Mount Lebanon, 240–960 mg/100 g in Bekaa, 80–520 mg/100g in Beirut, 252–1952 mg/100g in Tripoli, and 40–680 mg/100g in Saida.

One of the main factors of the richness of these Lebanese dishes in salt is mainly the high amount of salt added to the ingredients. Additional factors could be the use of spices rich in Na, like coriander leaf, cumin, cloves and cinnamon, during cooking, which are commonly used in the Lebanese cuisine ^22^. The Bahraini plates *Shawarma Lahme* and *Shawarma Djeij* contained 100 mg of Na ^23^, less than the Lebanese plates ( Table 3). Unfortunately, we could not compare the Lebanese traditional dishes to the traditional dishes in other Arab and Middle Eastern countries because few dishes are common between them.

### Iron

In general, meat-based dishes contained the highest levels of iron compared with other dishes. This was observed in Mount Lebanon particularly in *Chichbarak* and *Batata w kafta* (≥2.5 mg/100 g) ( Table 2). However, in all other governorates, the highest level of iron was shown in *Fatayer Sabanikh* (≥4.5 mg/100g) ( Table 2). As for the highest mean level of iron, Table 3 shows that *Fatayer Sabanikh and Kafta w Batata* ranked first. The Bahraini plates, *Shawarma Lahme* and *Shawarma Djeij*, contained the triple amount of iron (>3 mg) compared to the Lebanese plates (around 1 mg of iron) ^23^.

Given the available evidence about the influence of culture on health and health behaviors, reducing total sugar or salt and improving iron intake cannot be separated from cultural factors influencing nutrition-related-attitudes towards the health benefits of iron intake or the use of salt when cooking foods. Cultural influences play a critical role in framing how people perceive food preparation patterns, dietary habits, and coping with variability in agricultural conditions ^24^.

### Contributions to daily values

The nutrient goal represents the average intake that is compatible with maintaining of good health in individuals ^25^. According to the US Food and Drug Administration (FDA) definition, the daily value (DV) is "reference values for reporting nutrients on the nutrition labels". The percentage (%) DV assists the consumer in recognizing how the serving of food and its content in nutrients, fit into their daily diet ^25^. As per FDA regulations, the expression "high," "rich in," or "excellent source of" nutrients are used if the food has≥20% of the daily value per reference amount. The terms "good source," "contains," or "provides" are used if the food yields 10–19% of the Recommended Dietary Intake (RDI) per reference amount of the nutrient. Foods that carry <10% of the RDI from the nutrient per reference amount are considered as having low amounts ^25– 27^.

In our study, the contribution of each dish to the overall amount of iron, salt and total sugar needed per day was calculated. The calculations are presented in Table 4 and Table 5. The term "total carbohydrate" (CHO) includes dietary fiber, total sugar, added sugars and sugar alcohols. As per the updated FDA regulations, the DV of total CHO is 275 g/day (55% of energy intake in a 2000 kcal-diet) and the DV of added sugar is 50 g/day (10% of energy intake in a 2000 kcal-diet) ^26, 27^. As per Rapaille *et al*, and because of the low digestibility of polyols or sugar alcohols, the intake level is restricted to 40–50 g per day in adults ^28^. We hypothesized that based on the above recommendations; the daily total sugar consumption should not exceed 147 g per day. This number was obtained by subtracting 50g (recommendation of total sugar) and 50 g (recommendation of polyols) and 28 g (recommendation of fiber) from 275 g (recommendation of total carbohydrate). As per WHO, the iron intake should be limited to 18 mg/day ^15^ and the salt intake should not exceed 5g/day (1 tsp) ^29^.

**Table 4.** The percentage contribution to a 2000 Kcal diet of 100 g of traditional dishes for the amount of total sugar (Tot.S), salt (NaCl) and iron based on daily recommend amounts. The daily recommended amount of total sugar is 147 g; NaCl is <5 g;iron is18 mg.

Appetizer/Dish	Mount Lebanon			Bekaa			Beirut			Tripoli			Saida		
	Tot S.	NaCl	Iron	Tot S.	NaCl	Iron	Tot S.	NaCl	Iron	Tot S.	NaCl	Iron	Tot S.	NaCl	Iron
**Baba Ghanouj**	1.9	14	3.3	1.2	24	4.1	1.4	18	5.3	2	12.6	3.5	1.	18	4.1
**Batata mihchi**	1.7	22	6.3	0.2	16	6.2	1	14	3.9	1	23.8	6.6	0.5	26	7
**Bulgur b banadoura**	1	28	8.5	1	16	9.1	0.8	26	5.5	0.9	25	6.9	1.2	22	7.3
**Chichbarak**	1.5	14	15.7	1.8	22	15.2	1.7	14	4.3	4	22.8	6.3	1.7	20	14.3
**Falafel**	2.4	28	10.2	2.1	38	9.7	1.3	26	17.9	2.0	35	9.7	2.9	28	10.7
**Fatayer Sabanikh**	1	18	27.7	1.6	30	28.5	0.9	14	26.2	1.5	97.6	27.1	1.5	18	25.3
**Fatteh**	2	12	5.2	1.5	12	9.9	2.1	12	6.6	2.3	26.4	7.3	0.4	2	5.8
**Fattoush**	1	6	3.3	0.8	26	4.8	1.2	8	12.5	3.5	19	5.2	1.3	14	2.6
**Foul moudamas**	0.6	20	3.3	0.6	48	5.2	2.2	14	4.1	0.7	13.6	3.7	1.5	28	3.9
**Hindbe b zet**	2.8	14	9	0.8	18	11.1	1.5	16	14.6	3.1	34.4	9.5	0.4	18	9.9
**Hummus bi tahini**	1.0	16	4.7	1.8	12	6.6	1.6	16	6.6	2	20	5.5	1.2	18	5.2
**Kafta w batata**	0.7	24	22.9	0.6	18	7.2	1.3	16	7.9	1.2	25	6.9	0.8	24	22
**Kibbeh b sayniye**	1.7	24	11.3	1.3	20	11.6	2.2	16	7.5	1.7	37.4	10.3	0.8	22	10.8
**Koussa Mihchi**	0.2	24	7.3	0.5	18	6.5	0.7	14	9.8	1.2	35.2	9.7	0.5	24	6.7
**Lahm bi ajeen**	1	10	11	3.2	14	17.5	2.3	14	9.7	2.7	26.4	7.3	2.1	14	11.2
**Loubieh b zet**	1.2	12	4.8	0.4	16	6.5	1.2	18	5.1	0.6	41	11.3	0.6	12	4.3
**Malfouf mehche**	1.4	36	6	1	14	6	1.4	18	6.2	1.7	54.4	15.1	1	34	6.3
**Moujadara**	1	8	6.9	0.6	18	6.3	1.3	10	7.8	0.7	33.2	9.2	1	12	7.6
**Moghrabieh**	0.8	8	5.2	1	20	6.7	0.8	4	5.9	1.1	18.2	5	1.2	12	4.9
**Mousaka batinjen**	1.3	12	5.7	1	38	13.5	0.3	26	5.1	1.2	22.8	6.3	1.2	14	5.4
**Riz a djeij**	0.8	14	5.9	0.4	14	21.3	0.2	20	6	0.1	25	6.9	0.8	8	5.4
**Riz b lahme**	0.6	8	7.3	0.4	18	9.9	0.8	18	5.9	1.3	21.8	6	0.6	20	7.1
**Sayadieh**	0.2	16	7.3	0.5	30	7.8	0.1	16	3.9	0.4	25	6.9	0.4	28	6.6
**Shawarma Djeij**	1.5	28	9.2	1.3	24	8.8	1.2	16	8.7	1.2	27.2	7.5	0	16	8.8
**Shawarma Lahme**	0.6	14	8.3	0.6	28	7.9	0.7	8	7.6	0.7	35	9.7	0.6	18	7.6
**Tabboule**	0.4	22	6	0.8	38	6.7	0.9	12	13.2	0.8	30	8.3	0.5	14	5.4
**Warak enab**	0.7	26	7.3	0.6	18	9.7	1.7	14	5.9	0.5	20	5.5	0.2	26	7.8
**Yakhnet Bemieh**	0.9	22	8.6	3.7	16	5.9	1.1	18	7.7	1.5	30	8.3	1	14	8.2
**Yakhnet Fassoulia**	0.6	20	5.2	0.6	14	6.1	0.6	12	10.2	0.7	33.4	9.2	0.6	18	4.9
**Yakhnet Mloukhie**	0.6	18	5.8	0.5	18	6.5	0.1	14	5.7	0.6	33.4	9.2	0.5	18	6.5

We determined the percent contribution of 100 g of each traditional dish to the mean daily need of total sugar, salt, and iron in a 2000 Kcal-diet. The percentage of contribution of nutrients and minerals in a 2000 kcal-diet tested in 100 g of the dishes is shown in Table 4. Our data showed that all the traditional dishes in the five governorates contain low amount of total sugar (<10% of the DV). As for the salt, the majority (67%) of the dishes contains a high amount of salt; however this differed between governorates. A total of 43% of the dishes from Mount Lebanon and Bekaa, 13% of dishes from Beirut, 86% of the dishes from Tripoli, and 40% of dishes from Saida contained a high amount of salt (>20% of the DV). High sodium intake is associated with hypertension, a modifiable risk factor for CVDs ^5^. In 2013, Powles *et al*. estimated that 3.13 g per person per day was the daily sodium intake in the Lebanese population ^30^. In addition, a representative survey on 2,543 Lebanese adults showed that the average sodium intake was 2.9 g/day, with high intake in men (3.4 g/day) compared to women (2.4 g/day) ^31^. Around 60% of the adults in Lebanon are considered as exceeding the WHO limit level of Na intake of 2 g/day ^31^. Parallel to these alarming results, another study was conducted to investigate the knowledge, attitudes, and salt-related behaviors in Lebanese consumers. It showed that the majority of Lebanese participants developed discernible behaviors that increase the intake of salt and had limited knowledge of the food sources of salt ^22^. Thus, the adoption of long-term strategies by healthcare providers is required to control adding salt to reduce its negative effects, in Lebanon and elsewhere.

As for the iron content in the Lebanese traditional dishes, more than 60% of the recipes are deficient in iron (<10% of the DV of iron). Only 6% of the dishes from Mount Lebanon and Bekaa, and 3% of the dishes from Beirut, Tripoli, and Saida contain a high amount of iron in 100 g from each dish. Furthermore, in Table 5, the range of the percentage of DC of iron fluctuates between 4% and 27% indicating low content of iron in these dishes. Pellet and Shadarevian tested some of the same traditional dishes in 1970; their data also showed that all the dishes were also deficient in iron ^32^. More than one third of the region’s population suffers from anemia especially preschool children, pregnant women and women of childbearing age ^15^. Iron deficiency and iron-deficiency anemia have numerous adverse health consequences that affect the health of high-risk groups (women and children) ^15, 33^. Iron deficiency and iron deficiency anemia can be caused by numerous factors such as low iron intake, decreased iron absorption, chronic blood loss, and chronic inflammation ^15, 33^. There is a high worldwide prevalence range of iron deficiency anemia among pregnant women (14–75%) ^33^. This type of anemia can lead to maternal complications and increased the risk of obstetric hemorrhage in pregnant women ^34^. Furthermore, more than 50% of Lebanese hospitalized children aged between 1 month and 12 years, were anemic moderately or severely ^35^. Taking into consideration that anemia is prevalent in children and women, preventive measures related to good nutritional patterns and supplementation of iron-rich food are recommended. Strategies to tackle iron deficiency involve nutrition education and food fortification as well as targeted interventions for high risk groups, when needed. Wheat and maize flour fortification is a simple, inexpensive and effective strategy for supplying vitamins and minerals ^36^.

**Table 5.** The percentage contribution to a 2000 Kcaldiet of mean values of traditional dishes from five governorates for the amount of total sugar, salt and iron based on daily recommend amounts. The daily recommended amount of total sugar is 147 g; NaCl is >5 g; iron is 18 mg.

Appetizer/Dish	Percentage of daily contribution to 2000 Kcal-diet			
	Total sugar	Salt	Iron
**Baba Ghanouj**	1.5	17.3	4.1
**Batata mihchi**	0.9	20.3	6
**Bulgur b banadoura**	1	23.4	7.5
**Chichbarak**	2.1	18.5	11.2
**Falafel**	2.1	31	11.6
**Fatayer Sabanikh**	1.3	35.5	27
**Fatteh**	1.6	12.8	7
**Fattoush**	1.6	14.6	5.7
**Foul moudamas**	1.1	24.7	4
**Hindbe b zet**	1.7	20	10.8
**Hummus bi tahini**	1.5	16.4	5.7
**Kafta w batata**	0.9	21.4	13.4
**Kibbeh b sayniye**	1.5	23.8	10.3
**Koussa Mihchi**	0.6	23	8
**Lahm bi ajeen**	2.3	15.6	11.3
**Loubieh b zet**	0.8	19.8	6.4
**Malfouf mehche**	1.3	31.2	7.9
**Moujadara**	0.9	16.2	7.6
**Moghrabieh**	1	12.4	5.5
**Mousaka batinjen**	1	22.5	7.2
**Riz a djeij**	0.5	16.2	9.1
**Riz b lahme**	0.7	17.1	7.3
**Sayadieh**	0.3	23	6.5
**Shawarma Djeij**	1	22.2	8.6
**Shawarma Lahme**	0.6	20.6	8.2
**Tabboule**	0.7	23.2	7.9
**Warakenab**	0.7	20.8	7.3
**Yakhnet Bemieh**	1.6	20	7.7
**Yakhnet Fassoulia**	0.6	19.4	7.1
**Yakhnet Mloukhie**	0.4	20.2	6.7

There have been widespread priority actions that were implemented by the WHO Regional Office for the Eastern Mediterranean Region in response to unhealthy eating patterns among this population and the high prevalence of obesity. In 2016, taking into account the recommendations made by the WHO’s Commission on Ending Childhood Obesity, the WHO Regional Office for the Eastern Mediterranean Region prioritized the importance of dietary patterns modification at a regional level to halt the rise of NCDs in all age categories ^37^. These priority targets and interventions have sufficiently strong evidence based expert analysis ^38^. Furthermore, the WHO Regional Committee for the Eastern Mediterranean Region translated the United Nations political declarations and recommendations into a regional framework for action targeting the endorsement of population and individual measures by WHO Member States ^39^.

## Conclusions

At the national government level, all the related-health sectors should develop a comprehensive range of policy actions that provide support of healthy diet adherence to ensure that agriculture and food policies meet health recommendations. They should also assess the effects of the agri-food systems on NCDs. At the research level, academia should be engaged proactively in building evidence based, and communicating this data to policymakers, handling and supporting the development of data systems with research donors to enable countries to monitor the implementation of effective policies in conjunction with international health agencies.

The main limitation of this study is the analysis of nutrients and minerals in limited numbers of Lebanese traditional dishes. Furthermore, since the food samples were purchased from central kitchens, the proportions of ingredients were not described. The ingredients were retrieved from the literature ( Table 1). In addition, variations in ingredients and preparation methods among traditional dishes from various governorates in Lebanon were inevitable.

In conclusion, this study is a stepping stone for studies exploring the total sugar, salt and iron content of traditional dishes, as well as potential intake in the Lebanese population. It emphasizes the need for multi-cultural education and awareness on salt and iron and their impact on health because nutrition education has the power to productively modify behaviors, attitudes and consumption. Healthy choices result from empowering and motivating people by increasing their knowledge. In general, food composition data are essential for taking action to educate and inform the public about nutrition. There remains a need to expand implementation of rules on nutrition labeling and to scale up introduction of simplified front-of-pack nutrition labeling. Widespread adoption of nutrition policies and/or strategies, along with implementation of multisectoral coordination mechanisms, is indicative of strengthened political commitment to improve nutrition in the countries of the Eastern Mediterranean Region.

## Data availability

All data underlying the results are available as part of the article and no additional source data are required.

## References

[1] Noncommunicable diseases progresses monitor 2020.Geneva: World Health Organization;2020 Reference Source

[2] Popkin B Adair L Wen Ng S: NOW AND THEN: The Global Nutrition Transition: The Pandemic of Obesity in Developing Countries. *Nutr Rev.* 2012;70(1):3–21. 10.1111/j.1753-4887.2011.00456.x 22221213 PMC3257829

[3] Goryakin Y Rocco L Suhrcke M: The contribution of urbanization to non-communicable diseases: evidence from 173 countries from 1980 to 2008, *Econ Hum Biol.* 2017;26;151–163. 10.1016/j.ehb.2017.03.004 28410489

[4] Al Jawaldeh A Rafii B Nasreddine L: Salt Intake Reduction Strategies in the Eastern Mediterranean Region. *East Mediterr Health J.* 2019;24(12):1172–1180. 10.26719/emhj.18.006 30799557

[5] https://www.who.int/en/news-room/fact-sheets/detail/cardiovascular-diseases-(cvds)/accessed on June 10, 2020.

[6] https://www.who.int/cardiovascular_diseases/about_cvd/en/accessed on June 10, 2020.

[7] Unger T Borghi C Charchar F: 2020 International Society of Hypertension Global Hypertension Practice Guidelines. *J Hypertens.* 2020;38(6):982–1004. 10.1097/HJH.0000000000002453 32371787

[8] https://www.who.int/dietphysicalactivity/reducingsalt/en/accessed on July 28 2020.

[9] Liping H Kathy T Sohei Y: Effect of dose and duration of reduction in dietary sodium on blood pressure levels: systematic review and meta-analysis of randomized trials. *BMJ.* 2020;368:315. 10.1136/bmj.m315 32094151 PMC7190039

[10] Dinu M Pagliai G Casini A: Mediterranean diet and multiple health outcomes: an umbrella review of meta-analyses of observational studies and randomized trials. *Eur J Clin Nutr.* 2017;72(1):30-43. 10.1038/ejcn.2017.58 28488692

[11] https://www.tentamus.com/fda-nutrition-fact-panel-added-sugars/?cn-reloaded=1&cn-reloaded=1/accessed on June 10, 2020.

[12] Guideline: Sugars intake for adults and children.Geneva: World Health Organization;2015. 25905159

[13] https://www.fda.gov/food/new-nutrition-facts-label/how-understand-and-use-nutrition-facts-label/accessedon June 10, 2020.

[14] Global Action Plan for the prevention and control of non-communicable diseases 2013-2020.Geneva. World Health Organization.2013 Reference Source

[15] https://www.who.int/nutrition/publications/micronutrients/anaemia_iron_deficiency/9789241596107/en//accessed on June 10, 2020.

[16] Montagnese C Santarpia L Iavarone F: Food-Based Dietary Guidelines around the World: Eastern Mediterranean and Middle Eastern Countries. *Nutrients.* 2019;11(6):1325. 10.3390/nu11061325 31200446 PMC6627223

[17] Promoting a healthy diet for the WHO Eastern Mediterranean Region: user-friendly guide 2012 and the Lebanese FBDG. 2012 Reference Source

[18] Issa C Salameh P Batal M: The Nutrient Profile of Traditional Lebanese Composite Dishes: Comparison with Composite Dishes Consumed in France. *Int J Food Sci Nutr.* 2009;60(Suppl 4):285–95. 10.1080/09637480903107700 19657847

[19] Batal M Al-Hakimi A Pelat F: Dietary Diversity in Lebanon and Yemen: A Tale of Two Countries.Lebanon: American University of Beirut;2005.

[20] Greenfield H Southgate DAT: Food Composition Data Production Management and Use.2 ^nd^edition. Rome: Food and Agriculture Organization of the United Nations;2003 10.1007/978-1-4615-3544-7

[21] Association of Official Analytical Chemists: Official Method of Analysis.AOAC, Washington (DC), 19th edition,2012 Reference Source

[22] Nasreddine L Akl C Al-Shaar L: Consumer Knowledge, Attitudes and Salt-Related Behavior in the Middle-East: The Case of Lebanon. *Nutrients.* 2014;6(11):5079–5102. 10.3390/nu6115079 25401502 PMC4245581

[23] Musaiger AO: Food composition tables for kingdom of Bahrain. 1 ^st^edition. Bahrain: Arab Center for Nutrition. INFOODS regional database center;2011 Reference Source

[24] 2020 Global Nutrition Report: Action on equity to end malnutrition. Bristol, UK: Development Initiatives. ISBN: 978-1-9164452-7-7. Reference Source

[25] https://www.fda.gov/regulatory-information/search-fda-guidance-documents/industry-resources-changes-nutrition-facts-label/accessed on June 10,2020.

[26] https://www.fda.gov/food/food-labeling-nutrition/label-claims-conventional-foods-and-dietary-supplements/accessed on June 10, 2020.

[27] https://www.accessdata.fda.gov/scripts/cdrh/cfdocs/cfcfr/CFRSearch.cfm?fr=101.13/accessed on June 10, 2020.

[28] Rapaille A Goosens J Heume M: Sugar Alcohols. *Encyclopedia of Food Sciences and Nutrition.* 2003;5665-5671. 10.1016/b0-12-227055-x/01164-0

[29] WHO: Guideline: Sodium intake for adults and children. Geneva, World Health Organization (WHO),2012 Reference Source 23658998

[30] Powles J Fahimi S Micha R: Global, regional and national sodium intakes in 1990 and 2010: a systematic analysis of 24 h urinary sodium excretion and dietary surveys worldwide. *BMJ Open.* 2013;3(12):e003733. 10.1136/bmjopen-2013-003733 24366578 PMC3884590

[31] Lebanese Action on Sodium and Health. accessed on June 12, 2020. Reference Source

[32] Pellett P Shadarevian S: Food composition tables for use in the Middle East. 2 ^nd^edition. Beirut, American University of Beirut,1970 Reference Source

[33] Mirza F Abdul Kadir R Breymann C: Impact and management of iron deficiency and iron deficiency anemia in women's health. *Expert Rev Hematol.* 2018:11(9):727–736. 10.1080/17474086.2018.1502081 30019973

[34] Khalafallah A Dennis AE: Iron deficiency anaemia in pregnancy and postpartum: pathophysiology and effect of oral versus intravenous iron therapy. *J Pregnancy.* 2012;2012:630519. 10.1155/2012/630519 22792466 PMC3389687

[35] Salami A Bahmad HF Ghssein G: Prevalence of anemia among Lebanese hospitalized children: Risk and protective factors. *PLoS One.* 2018;13(8):e0201806. 10.1371/journal.pone.0201806 30086152 PMC6080804

[36] WHO: Recommendations on wheat and maize flour fortification: meeting report-interim consensus statement. Geneva, world Health Organization;2009 Reference Source 24809114

[37] Report of the Commission on Ending Childhood Obesity. Geneva: World Health Organization;2016.

[38] https://extranet.who.int/iris/restricted/bitstream/handle/10665/259519/emropub_2017_20141.pdf;jsessionid=669A113AC9CBCD95934ECDB66AEAFF1A?sequence=1/acced on July 28, 2020.

[39] Framework for action to implement the United Nations Political Declaration on Noncommunicable Diseases. Cairo: World Health Organization Regional Office for the Eastern Mediterranean;2015 Reference Source

