# Supplementary material for: Nutritional value of the Middle Eastern diet: analysis of total sugar, salt, and iron in Lebanese traditional dishes
Source: F1000Res. 2020 Oct 19;9:1254. [Version 1] doi: 10.12688/f1000research.26278.1 (PMC7702163; doi:10.12688/f1000research.26278.1)
Supplement: Peer review history

# Peer review: Reviewer response for version 1

**DOI:** 10.5256/f1000research.29005.r73820

**Published:** 2020-11-26

**Authors:** Haleama Al Sabbah

**Affiliations:** 1 Zayed University, Dubai, United Arab Emirates

Thanks to the journal and authors for giving me this opportunity to review this excellent work.

 The main objective of this study is to establish a database analysis of total sugar, salt and iron content in Lebanese foods, focusing on traditional dishes. To achieve this objective, the researchers collected the data by using a stratified sampling methodology. The number of samples per governorate was estimated to be 30 according to the variability in the dishes' composition. The presented results are mapped with the objective and assess the total sugar, sodium (Salt) and iron.

 In order to improve the work of this paper I have the following comments for the authors:

**General**

 There is a need for language editing and proof reading for the consistency in writing and phrasing some of the Arabic food names.

 The list of References at the end of the paper needs to be checked, delete the repeated references and be consistent in the style of referencing. The majority the references are links to websites and when trying to open some of these links it says that the page is not found (e.g. https://www.fda.gov/regulatory-information/search-fda-guidance-documents/industry-resources-changes-nutrition-facts-label/ accessed on June 10, 2020).

**Detailed comments:**

**Abstract:**

***“ ...*** *food samples was done using stratified sampling techniques. These samples were classified into five strata, taking into account variation by geographical area (Mount Lebanon, Bekaa, Beirut, Tripoli, and Saida)* *”* *.*

 The above statement needs rephrasing as by reading further in the methodology and sampling, it’s clear that the sampling methodology is convenience sampling technique from five regions/ governorate and not a stratified method.

 It is also not clear in the abstract if the analysis of the samples had been done theoretically or in the lab? You need to add the software used to analyse the data at the end of the methods section. Also, the study design and setting should be added to the methods of the abstract. The total sample size?

 The following statement needs re-phrasing “... little total sugar” how you define little?” - What is the number/percentage?

**Main Manuscript:**

**Methods:**

 Need to be consistent with the methods presented under the abstract and in the main body of the manuscript (e.g. sampling methodology *?) “* *Our research group collected 500 g of 30 types of traditional dishes from five different central kitchens in the following governorates* *”*

 How many kitchen from each region they used? What are the selection criteria for the kitchen where you take the sample from? Do they know that you are analyzing their food contents for sugar, iron and salt? If they knew that do you think they did changes in the recipe/ ingredients for each dish of food? 

 It’s not clear enough how did you do the analysis in the lab for each nutrient.

*“* *All the above chemical analyses were conducted at the Industrial Research Institute (Beirut, Lebanon), which is an accredited laboratory for chemical analyses. After food samples were analyzed, stamped laboratory results were received and data was entered on Excel 2019. The data validation process was conducted, and cross checked by two research assistants.* *”*

 The above paragraph should be revised and integrated with more information about the study design, settings and sampling methodology.

 It's better to move Table 1 to the annex with more specific and quantifiable ingredients of the recipes for each kitchen you have selected and have samples from.

**Results**

**Total Sugar**

*“* *Among all the governorates, all the tested traditional Lebanese dishes contained little total sugar.* *”*

 What is the definition for 'little total sugar'? How did you judge that it is little and according to what? You need a reference to compare and say that it is little.

 Table 3, the mean values in 100g of food is not clear if it’s in gram, milligram or microgram? The unit should be written under each item.

**Salt**

 In the statements under salt “ *The sodium (Na) level was high in most dishes (Table 2)*.” and “ *One of the main factors of the richness of these Lebanese dishes in salt is mainly the high amount of salt added to the ingredients.* *”*

 How did you judge that the level is high and not low or medium? What is your reference for that and compared to what? You need to have a reference in order to compare and say that it's high otherwise it should be descriptive only without analytical statement that its high.

 The titles of Table 4 & 5 are too long and some of the information can be inserted in the tables heading or adding them under the table as key information.

Is the work clearly and accurately presented and does it cite the current literature?

Yes

If applicable, is the statistical analysis and its interpretation appropriate?

Yes

Are all the source data underlying the results available to ensure full reproducibility?

Yes

Is the study design appropriate and is the work technically sound?

Yes

Are the conclusions drawn adequately supported by the results?

Yes

Are sufficient details of methods and analysis provided to allow replication by others?

Yes

Reviewer Expertise:

Public Health and Nutrition

I confirm that I have read this submission and believe that I have an appropriate level of expertise to confirm that it is of an acceptable scientific standard.

# Peer review: Reviewer response for version 1

**DOI:** 10.5256/f1000research.29005.r73294

**Published:** 2020-11-02

**Authors:** Reema Fayez Tayyem

**Affiliations:** 1 Department of Nutrition and Food Technology, Faculty of Agriculture, University of Jordan, Amman, Jordan

It is a well-written and informative manuscript. This study is targeted to establish and initiate a database analysis of total sugar, salt, and iron content in Lebanese foods, focusing on traditional dishes.

 A few suggestions and corrections have been addressed to improve the quality of the paper.

 All these corrections are presented in the pdf file attached.

Is the work clearly and accurately presented and does it cite the current literature?

Yes

If applicable, is the statistical analysis and its interpretation appropriate?

Not applicable

Are all the source data underlying the results available to ensure full reproducibility?

Yes

Is the study design appropriate and is the work technically sound?

Yes

Are the conclusions drawn adequately supported by the results?

Yes

Are sufficient details of methods and analysis provided to allow replication by others?

Yes

Reviewer Expertise:

Human Nutrition and policy planning.

I confirm that I have read this submission and believe that I have an appropriate level of expertise to confirm that it is of an acceptable scientific standard.
